# Health Benefits of Plant-Derived Sulfur Compounds, Glucosinolates, and Organosulfur Compounds

**DOI:** 10.3390/molecules25173804

**Published:** 2020-08-21

**Authors:** Natalia Miękus, Krystian Marszałek, Magdalena Podlacha, Aamir Iqbal, Czesław Puchalski, Artur H. Świergiel

**Affiliations:** 1 Department of Pharmaceutical Chemistry, Faculty of Pharmacy, Medical University of Gdańsk, Hallera 107, 80-416 Gdańsk, Poland; natalia.miekus-purwin@gumed.edu.pl; 2 Department of Fruit and Vegetable Product Technology, Prof. Wacław Dąbrowski Institute of Agricultural and Food Biotechnology, 36 Rakowiecka, 02-532 Warsaw, Poland; 3 Department of Food Technology and Human Nutrition, Institute of Food Technology and Nutrition, College of Natural Science, University of Rzeszow, Zelwerowicza 2D, 35-601 Rzeszow, Poland; 4 Department of Molecular Biology, Faculty of Biology, University of Gdańsk, Wita Stwosza 59, 80-308 Gdańsk, Poland; magdalena.podlacha@ug.edu.pl; 5 College of Food Science and Technology, Huazhong Agricultural University, Wuhan 430070, China; aamirraoiqbal@yahoo.com; 6 Department of Bioenergetics and Food Analysis, Faculty of Bogy and Agriculture, University of Rzeszow, Ćwiklińskiej 2D, 35-601 Rzeszow, Poland; cpuchal@ur.edu.pl; 7 Department of Animal and Human Physiology, Faculty of Biology, University of Gdańsk, Wita Stwosza 59, 80-308 Gdańsk, Poland; swiergiel@yahoo.com; 8 Prof. Wacław Dąbrowski Institute of Agricultural and Food Biotechnology, 36 Rakowiecka, 02-532 Warsaw, Poland

**Keywords:** alliaceous vegetables, cruciferous vegetables, sulfur-based compounds, food processing, nutraceuticals, functional food

## Abstract

The broad spectrum of the mechanism of action of immune-boosting natural compounds as well as the complex nature of the food matrices make researching the health benefits of various food products a complicated task. Moreover, many routes are involved in the action of most natural compounds that lead to the inhibition of chronic inflammation, which results in a decrease in the ability to remove a pathogen asymptomatically and is connected to various pathological events, such as cancer. A number of cancers have been associated with inflammatory processes. The current review strives to answer the question of whether plant-derived sulfur compounds could be beneficial in cancer prevention and therapy. This review focuses on the two main sources of natural sulfur compounds: alliaceous and cruciferous vegetables. Through the presentation of scientific data which deal with the study of the chosen compounds in cancer (cell lines, animal models, and human studies), the discussion of food processing’s influence on immune-boosting food content is presented. Additionally, it is demonstrated that there is still a need to precisely demonstrate the bioavailability of sulfur-containing compounds from various types of functional food, since the inappropriate preparation of vegetables can significantly reduce the content of beneficial sulfur compounds. Additionally, there is an urgent need to carry out more epidemiological studies to reveal the benefits of several natural compounds in cancer prevention and therapy.

## 1. Introduction

Attempts to modulate the immune system started in the mid-1970s [1] with the introduction of bacterial toxins. The main disadvantages of those therapies were connected to regulatory problems, the impurity of the exogenic modulatory cocktail, or the occurrence of multiple side effects. However, the discoveries in the field of natural components of the food that could display immunomodulatory properties led to the recognition of novel natural compounds that could preserve the homeostasis of the human immune system. These natural therapies include natural plant-derived food compounds that have been proved to be beneficial over synthetic or uncontrolled (such as neutralized pathogens) compounds [2]. The natural immune boosters present a really promising approach for immunomodulation and could be beneficial over synthetic drugs due to their lower cost, decreased or even eliminated toxicity, and few adverse effects. The study of natural immune boosters is relevant also because the imbalance in the immune system is involved in the pathogenesis and progression of several serious diseases, such as infectious and autoimmune diseases, allergies, obesity, metabolic syndrome, cardiovascular disorders, gastric ulcers, and cancer [3,4]. Nutraceuticals can exert indirect antitumor effects via immune enhancement [5]. In terms of cancer, an up to 40% decrease in cancer cases could be gained just via the administration of proper food and nutrition [6]. A connection between inflammation and cancer has been recognized since the 17th century, and over 150 years ago we were provided with a scientific rationale by Robert Virchow [7]. A number of cancers have been associated with inflammatory processes. This now has some implications for their prevention and treatment [8]. Indeed, there is solid evidence that the increased risk of malignancy is associated with the chronic inflammation caused by chemical and physical agents and different inflammatory reactions involving leucocytes and tumor cells. The focus has been on cytokines and chemokines; their expression; their mechanisms of action; and, consequently, their preventive and therapeutic targeting of inflammation [9]. The basic interactions between different nutrients, including those of plant origin; pro-inflammatory cytokines; and inflammation have been succinctly presented by Grimble [10,11]. In the context of the current review, the obvious question is whether plant-derived sulfur compounds can be beneficial for cancer treatment as a supportive therapy. Numerous plant compounds (saponins, alkaloids, phenolics, triterpenes, coumarins, polysaccharides, anthraquinones, etc.) exert antitumor properties, not only by a direct toxic effect on malignant cells but also by influencing macrophage phenotypic differentiation and, possibly, cytokine secretion [12]. Among them, the sulfur compounds found in the alliaceous and cruciferous families were studied for their potential in cancer treatment. 

Cruciferous vegetables including broccoli (*Brassica oleracea var. italica*), cabbage (*Brassica oleracea var. capitata*), cauliflower (*Brassica oleracea var. botrytis*), and other plants of the family brassicaceae are rich sources of bioactive compounds, including vitamin C and E, dietary fiber, and the glycosides of the flavonoids quercetin and kaempferol. However, the components that make cruciferous vegetables so beneficial to human health and set them apart from other plants are the sulfur-containing glycosides (glucosinolates, GSLs). Dietary GSLs are considered as potential anticancer agents [13]. The bioactive substances found in cruciferous vegetables can inhibit inflammation by activating detoxification enzymes, clearing free radicals, and inducing immune functions.

Onion (*Allium cepa*) and garlic (*Allium sativa*) are some of the oldest cultivated plants in the world and are rich in flavonols and natural organosulfur compounds (OSCs). Their consumption was studied, as it was considered beneficial for heart diseases, tumors (such as esophageal, gastric, colorectal, breast, lung, skin and prostate cancers), and immunological processes, among others, especially due to the biological action of the sulfur-containing metabolic byproducts [14,15,16]. They act by the induction of phase II detoxification enzymes [17], take part in the scavenging of free radicals, and change the morphology of the cells that abort cell-to-cell contact [18]—effects that all together are known to contribute to cancer inhibition. On the other hand, the side effects of the excessive consumption of these plants have been described. Since the studies have been conducted mainly in animal models, the need for the full characterization of the proper dosages of OSC consumption remains an important task for further study [19]. Moreover, the need to precisely demonstrate the bioavailability of sulfur-containing compounds from various types of functional food remains a challenge. 

The present review aims to characterize the general characteristics, physicochemical properties, and health-related studies of onion, garlic, and cruciferous vegetable consumption, as well as the influence of food processing on their content of active substances. The review combines distinct chapters where information relevant for nutraceutical and functional food engineers, medical doctors, as well as consumers can be obtained. We believe that the review will stimulate further studies related to the implementation of the most accurate sources of sulfur compounds from chosen plants which could support the therapy of cancer patients. Moreover, the review aims to present cost-effective anti-tumor means that would include the implementation in the diet of sulfur-based compounds in foods. 

## 2. Plant-Derived Sulfur Compounds—GSLs and OSCs 

### 2.1. Glucosinolates (GSLs)

Sulfur is an essential compound for the biosynthesis of various secondary metabolites, such as phytoalexins, GSLs, and alliins. These metabolic compounds play a vital role in the physiology and protection of plants against several environmental stresses [20]. More than 200 different types of GSLs have been studied, and 30 of them are identified in Brassica crops [20,21]. GSLs are major secondary metabolites synthesized by plants. GSLs are present in various species of the Brassica family. These are responsible for the nutritional characteristics of Brassica products, such as vegetables, oil, and meal [22]. Moreover, they are also responsible for the flavor and taste of Brassica products. The breakdown products of GSLs play a vital role in plant defense mechanisms against pathogens and insects. In addition, these breakdown products have gained much attention because of their anti-carcinogenic properties [23].

#### 2.1.1. Chemical Structure and Biodegradation

**Figure 1:**
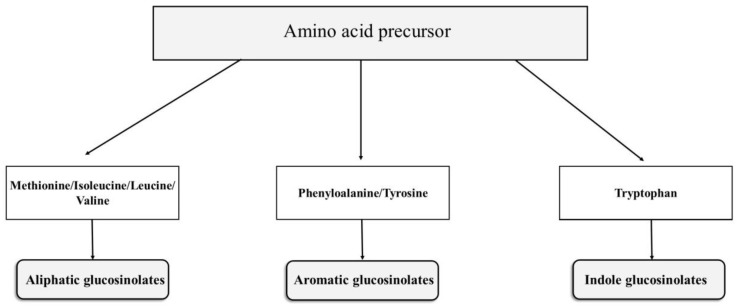
Glucosinolate classification based on the structure of their amino acid precursors. Among the common amino acid precursors of glucosinolates are aromatic amino acids, phenylalanine, or tyrosine; aliphatic amino acids include alanine, leucine, isoleucine, methionine, and valine, whereas indole glucosinolates are those derived from tryptophan.

GSLs are organic anions molecules comprised of β-D-thioglucoside linked to the (Z)-N-hydroximinosulfate group and a variable side chain derived from a variable amino acid R-group [24]. GSLs molecules are synthesized by the action of different chemical compounds, including two cytochromes P450, glucosyl transferase, C-S lyase, and sulfotransferase. GSLs can be divided into three classes—aliphatic, indole, and aromatic—which are derived from amino acid precursors such as methionine, tryptophan, and phenylalanine, respectively (Figure 1) [25,26]. GSLs with an aliphatic side chain derived from methionine are most prominent in the leaves of Brassica vegetables. Aliphatic GSLs are classified by the size of carbon side chains, such as 3-carbon, 4-carbon, and 5-carbon GSLs. They can also be classified by the structure of the side-chain, such as hydroxyalkeny, l methylsulfinylalkyl, methylsulfanylalkyl or methylsulfonylalkyl glucosinolates [27].

**Figure 2:**
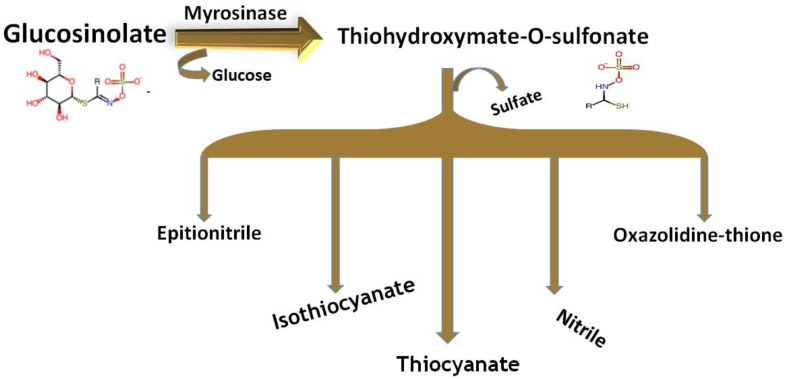
Breakdown of glucosinolates and the possible alternative breakdown products.

GSLs are relatively stable compounds, but plant cells containing glucosinolates can be damaged due to the release of β-thioglucosidase or myrosinase enzymes during the cutting, mixing, chopping, and chewing of food (Figure 2). This enzyme is usually stored separately from GSLs, in different cells depending on the plant species [28,29]. The hydrolysis of GSLs by the enzyme myrosinase yields a β-D glucose molecule and an unstable aglycone (thiohydroximate-*O*-sulfonate). Upon the cellular injury of plant tissues, GSLs are enzymatically degraded by myrosinase (a thioglucosidase) to produce a variety of different breakdown products, such as glucose, sulfate, isothiocyanates, epithionitriles, nitriles, indolic alcohols, oxazolidinethions, amines, and thiocyanate. It is believed that both GSLs and their breakdown products play an important role in the defense mechanism of plants against the attacks of fungi, insects, and microorganisms [23,30]. Moreover, these are also presumed to have different health-promoting roles, which makes them beneficial compounds for human consumption. 

In order to preserve the beneficial properties of cruciferous plants, it is important to store and prepare them properly. The content of glucosinolates varies between cruciferous plant types, but the bioavailability of these compounds can be significantly increased by appropriate storage and preparation. The requirements for storage conditions vary in the available literature. Some reports indicate long-term storage at room temperature and in the refrigerator. Others suggest a significant decrease in GSLs after seven days of storage. An even greater decrease was noted after 6 h of storage for cruciferous vegetables that were previously chopped [31]. In the analyzed brassicaceae vegetables, the highest GSL content was found in purple cauliflower, while green cauliflower had the lowest content of those compounds [32]. However, the inappropriate preparation of cruciferous vegetables can significantly reduce the content of the mentioned compounds. Various processes used to prepare cruciferous vegetables for consumption, such as cutting, crushing, or cooking, disintegrate the cells and are a strong stress factor that stimulates the GSL hydrolysis reaction. As a result of boiling different brassicaceae vegetables for several minutes, a significant (36%) reduction in GSLs was observed. Therefore, in order to keep the content of the described compounds as high as possible, it is recommended to choose methods that require a small amount of water. GSL and its derivatives in cruciferous vegetables have a high potential for use as functional food. However, high-temperature cooking practices such as boiling, frying, blanching, and high-power microwave processing can degrade the bioavailability of GSLs, vitamins, phenolics, and other health-promoting compounds in brassicaceae vegetables [33,34]. Many studies indicate that the most efficient method for processing these vegetables is steam cooking, as it preserves many nutrients and other compounds beneficial to human health, including GSLs [35].

Several researchers have studied the thermostability and degradation of the GSLs contained in broccoli, red and white cabbage, and brussel sprouts. The results obtained indicate that the important determining factor is media type (food matrix or cooking water) [36]. Florkiewicz et al. [37] suggested sous-vide as the most appropriate and beneficial method of preparing cruciferous vegetables. Inappropriate thermal processing causes the degradation not only of GSLs, but also of indoles and other isothiocyanates. In turn, the research of Kapusta-Duch et al. [38] has shown a very high level (48.5; 75.8%) of indole decomposition as a result of boiling for 15 min at 100 °C. Under the same conditions, isothiocyanates (11.0; 42.4%) as well as ascorbic acid (33.24; 16%) also degrade significantly [39]. Whereas, Westphal et al. [40] evaluated the impact of high-pressure processing (HPP) on GSLs and their breakdown products. For instance, the activity of myrosinase, an enzyme which hydrolyzes GSLs and improves beneficial health effects, was not impaired in HPP-treated vegetables. However, the level of conversion was highly dependent on the parameters used. Additionally, the UV light seems to be not without significance as far as the activity of enzymes crucial for GSL metabolism is concerned [41]. The UV light stimulates, among other things, peroxidase activity, and affects the metabolism of flavonoids and promotes the antioxidant capacity of different cruciferous plants. Formica-Oliveira et al. [42] have confirmed that the use of UV radiation (2.2, 8.8, and 16.4 kJ/m^2^/day) has a positive effect on the content of GSLs in broccoli. One of the newer and more promising methods that can be used in the treatment of cruciferous vegetables while maintaining a high GSL content is pulsed electric field (PEF). The advantage of this technique is the possibility to penetrate into the plant tissue without using a very high temperature and damaging the tissue mechanically [43]. With the proper selection of parameters, this method may prove extremely useful, although only a few reports confirm its beneficial impact on the content of GSLs and other compounds, as well as their derivatives.

An important factor determining the bioavailability of GSLs is also human microflora. The most crucial role from the point of view of GSL metabolism is played by such enzymes as myrosinase and β-thioglucosidase. β-thioglucosidase is present in the human gut microbiome and influences human physiology and health, for example by regulating the metabolism of GSLs and their derivatives. In turn, the human homolog of myrosinase has not been described so far. The direct administration of isothiocyanates results in their higher bioavailability than that of GSLs, which indicates fundamental differences in such properties as lipophilicity. Therefore, a detailed knowledge of the factors determining the efficiency of the GSL to isothiocyanates conversion seems to be crucial [28]. A common method for assessing bioavailability is to measure the concentration of a particular compound (for example, sulforaphane (SFN)) or its metabolites in urine. In one of the clinical trials, the volunteers were given SFN- or glucoraphanin (GR)-rich beverages, and then the level of metabolites in urine was studied. The level of metabolites tested was significantly higher in SFN recipients. Other studies carried out on a group of healthy volunteers in China showed a high rate of SFN metabolite removal. Studies were also conducted to compare the degree of absorption of particular compounds from fresh and processed food. Four healthy volunteers who were given 3-day-old broccoli sprout extract hydrolyzed by myrosinase, which showed high levels of isothiocyanates. Other researchers have confirmed significantly higher levels of SFN in urine and blood plasma after the ingestion of fresh broccoli sprouts than commercially available myrosinase-free dietary supplements rich in glucoraphanin. However, another clinical trial carried out on a small group of individuals showed a similar level of bioavailability both when taking a commercially available supplement rich in GR but free of myrosinase and frozen broccoli sprout extract, suggesting a large individual variation in the absorption of the compounds described. It was also found that the factor determining the bioavailability of GSLs is the presence of myrosinase. This was confirmed by studies comparing the level of SFN metabolites after the consumption of powder prepared from fresh broccoli rich in GSLs without myrosinase and with it, respectively. It can undoubtedly be deduced that the way cruciferous vegetables are stored and prepared is of key importance for the preservation of compounds beneficial to the proper functioning of the human organism, such as GSLs and their metabolites. On the basis of the described reports, it can be summarized that the consumption of raw cruciferous vegetables, which allows one to maintain a high activity of endogenous myrosinase, is the most appropriate from the point of view of the bioavailability of the analyzed compounds. Since the pharmacokinetics of GSLs are complex and depend on many factors, such as the type of food matrix, the temperature, and the type of vegetable processing, as well as type of starting product (raw or processed vegetable) and the individual differentiation of the influence of the intestinal microflora, further research is necessary to be able to fully explain their potential in many fields of science and especially medicine [44].

In addition to the scientific challenges, the creation of supplements from properly prepared cruciferous plants to increase the degree of GSL assimilation is a great opportunity, especially since heat treatment is one of the factors negatively affecting the degree of absorption. In the United States, there is a growing increase in the sale of dietary supplements from cruciferous plants, which are prepared in the form of extracts and powders and then sold in capsule form. This again confirms the need for a deeper analysis of many factors influencing the transformation and metabolism of the compounds they contain, not only in healthy people but also in the interactions with drugs taken or the possible therapeutic potential. The Food and Drug Administration (FDA) has also issued a number of warnings about the information on the labels of these types of dietary supplements that provide anti-cancer properties and other beneficial effects but which have not yet been confirmed by clinical trials [44]. This is particularly important given that not all the mechanisms that influence the sulfur compounds contained in cruciferous vegetables are fully understood.

#### 2.1.2. Metabolism Pathway of Glucosinolates in the Body

Currently, little is known about how GSLs and their derivatives are metabolized in the human organism, as most reports relate to cell culture and animal studies. Data in humans are extremely sparse and mostly relate to the level of metabolites in the urine. With many compounds, it is the case that, despite the high level of absorption, the bioavailability is limited and reduced due to the high metabolic rate. With regard to the literature data on GSL metabolites, they are most often the result of the level of nitrogen compounds, isothiocyanates, and other derivatives. Additionally, the measurement of the level of metabolites in urine confirms that the hydrolysis reaction is an important criterion for the bioavailability of GSLs. The level of metabolites resulting from prior hydrolysis was six times higher than that of GSLs [45]. The highest concentration and level of glutathione and glutathione s-transferase activity have been reported in the liver. An increased concentration of isothiocyanates results in a decrease in the glutathione activity. Even with isothiocyanates which are similar in structure, their half-lives can vary by up to several hours. The kidneys are the main organ in which the conversion of glutathione conjugates into mercapturic acids takes place. This reaction is catalyzed by γ-glutamyltransferase. There are also many indications that there is a relationship between the structure of the isothiocyanate side chain and the intensity of a particular metabolic pathway, which translates directly into metabolites in urine [46]. The bacterial microflora of the human gastrointestinal tract is also directly related to the metabolism of GSLs. In human feces were identified, for example, a strain of the Bacteroides thetaiotaomicron, which is capable of breaking down GSLs. The high level of bacterial myrosinase activity allows a significant increase in the efficiency of the GSL metabolism, which translates into a high level of their metabolites in the feces. The main factors determining the absorption of GSLs from the intestinal lumen are not endogenous enzymes, but myrosinase activity in the plant tissues, which also affects the absorption of nitriles into the circulation. In the absence of active myrosinase, some GSLs can be absorbed directly from the human digestive tract, but the significance of this pathway is not fully understood [47].

#### 2.1.3. Health Benefits of Glucosinolates

The phytochemicals in cruciferous plants can exert different effects that affect the immune system. Inflammation leads to a number of changes in the organism, such as increased cellular proliferation; the inhibition of apoptosis; and, as a result, an increased risk of various types of cancer. Biologically active substances that occur in cruciferous plants inhibit the development of inflammation by, for example, activating detoxification enzymes, removing reactive oxygen species, and stimulating natural defense mechanisms [31]. Specific compounds of certain vegetables of the family brassicaceae block NF-κB activation; this is done through various mechanisms, such as the inhibition of TNF-α, IκBα, and p65 protein, which also regulates the pro-inflammatory signaling pathway. This effect was primarily mediated by SFN.

In addition to those effects described above, SFN shows a wide range of impacts. It blocks apoptosis, inhibits the release of reactive oxygen species, and affects the expression of vascular cell adhesion molecule (VCAM)-1 or key molecules in the IL-1 signaling cascade. Other important properties of this isothiocyanate include the suppression of such inflammatory reaction markers as interferon regulatory factor 3 (IRF3), as well as macrophage migration inhibitory factor (MIF). These beneficial effects of SFN are the result of the activation of various signaling pathways and mechanisms, which involve, among other Toll-interleukin-1 receptor domains, Toll-like receptors (TLRs) and interferon-beta. Among the numerous activities of SFN, its chemopreventive potential cannot be overlooked. Indeed, SFN suppresses cyclooxygenase-2 (COX-2), which has immunoreactivity properties. These effects were again mainly NF-κB- and ERK (extracellular-signal-regulated kinase)-mediated. Another important compound of cruciferous vegetables with a significant impact on the immune system is berteroin (5 methylthiopentyl isothiocyanate). It is found in the largest quantities in vegetables, such as cabbage and rucola. It has been shown that its frequent consumption reduces edema by modulating COX-2, NF-κB, and ERK activity. There are also reports indicating the beneficial effect of cruciferous vegetables on the immune system parameters in healthy people. These concerns, in particular, the concentrations of circulating inflammatory biomarkers, such as IL-6, CRP, and sTNFRI. However, further more complex studies are needed to confirm that a cruciferous vegetable-rich diet reduces high levels of inflammatory markers in populations where this pathological condition persists chronically [48]. 

Glucosinolates and their metabolites, such as isothiocyanates like sulforaphane (SFN), have several health-promoting activities (Table 1). Sulfur-containing glycosides (glucoisnolates) are not biologically active but are hydrolyzed by myrosinases to indoles, thiocyanates, and isothiocyanates, which exhibit biological potential. Some isothiocyanates (for example, SFN is one of the best known isothiocyanates), are useful in stress response and anti-inflammation due to their inhibition of transcription regulator (NF-κB). Others play an important role in activating the synthesis of regulatory protein Nrf2, which promotes antioxidants and phase II microsomal enzymes [49].

For example, SFN is one of the best known isothiocyanates, mainly due to its anticancer properties, but also because of its ability to reduce the secretion of inflammation markers by immune system cells, as well as nuclear factor kappa B (NF-κB), which plays an important role in the rapid regulation of immune reactions. In vitro studies have confirmed the anti-inflammatory properties of a diet rich in cruciferous vegetables. Among others, reduced serum concentrations of pro-inflammatory cytokines, such as IL-1β, TNFα, and IL-6, were observed [50]. Several studies have confirmed that natural aryl hydrocarbon receptor (AhR) ligands are derived from food, such as broccoli. This AhR has been identified as one of the key immune receptors, and is expressed by not only different types of immune system cells, but also by certain types of cancer cells. The incorporation of the relevant ligands, including those found in cruciferous plants, results in activation, which transfers them to the nucleus. The resulting heterodimer exerts a number of different effects not only on the metabolic pathways, but also on genes whose expression modifies the course of immune reactions and the activity of various cytokines, including IL-22. The deficiency of the mentioned ligands has many negative consequences for the functioning of the whole organism. The digestive system is most exposed to these deficiencies, which manifest mainly in adverse effects on the bacterial intestinal flora, reduced granzyme production, and severe colitis [51]. Interestingly, mice studies have confirmed that a diet rich in cruciferous plants, which contain high levels of natural AhR ligands, mitigates these undesirable effects, as well as having a positive influence on the proper development of the immune system at an early stage of life. Isothionates can produce different compounds such as erucic and olic acids, which are considered to have strong antimicrobial effects. GSLs and their derivatives have also been reported to have chemopreventive actions [52]. 

##### Protection against Carcinogenesis 

The dietary consumption of GSLs and their metabolites may diminish the damaging impact of chemical carcinogenesis. A study reported that the risk of colon cancer is enhanced among individuals with a low consumption of broccoli, cabbage, and brussel sprouts, and reduced among those with a high consumption of these vegetables [66]. These findings suggested that GSLs and their metabolites may protect against environmental toxins by enhancing their detoxication. Isothiocyanates may also impart protection against carcinogenesis by arresting the cell cycle and inducing the apoptosis of cancer cells, thus reducing tumor growth. Moreover, a third mechanism is the modulation of epigenetic marks. Indeed, dietary SFN can inhibit the activity of histone deacetylase and increase the histone acetylation, enhancing protection against carcinogenesis [54,67]. In cancer, the strongest effect was discussed for the isothiocyanate SFN and its involvement in the induction of antioxidant pathways (through the regulation of the phase I and II detoxification enzymes, nicotinamide adenine dinucleotide phosphate (NADPH) regeneration, among others), and apoptosis in cancer cells, and its inflammation- and angiogenesis-inhibiting potential was revealed [55,56] for many cancers. A study of SFN in prostate cancer was conducted. The results obtained for the transgenic adenocarcinoma of the mouse prostate in a mice model indicated that SFN may play a role in the induction of the cytotoxicity of natural killer cells [68]. Further studies revealed that the prominent role of SFN in prostate cancer is the regulation of fatty acid synthesis; the active metabolite of SFN reduces the metabolism of fatty acids, which is beneficial for the primary and advanced stages of the disease [69]. Additionally, the involvement of SFN in the progression of prostate cancer has been studied. The data obtained showed that the inhibition of the deacetylation of histones is driven by SFN [70,71]. Not only preclinical studies but also studies in humans showed that, by taking SFN, a reduction in prostate cancer incidence and progression could be obtained [72,73]. The randomized double-blinded controlled trial named the “Effect of Sulforaphane on prostate CAncer PrEvention (ESCAPE)” demonstrated that taking 4-methylsulphinylbutyl glucosinolate (which is converted into SFN) for 12 months reduced the expression of genes that could be connected to the oncogenic pathway. The authors discussed that the main pathway of SFN action in prostate cancer patients was not directly focused on cancerous clones but more likely associated with the anti-aging effect of this phytochemical. Additionally, the epigenetic factors were discussed as the driving forces of GSLs’ anti-cancer activity [74]. A novel mechanism of chemoprotection by SFN was also proposed several years ago. Myzak et al. [75] proved that SFN takes part in the inhibition of histone deacetylase (HDAC) activity, the expression of its proteins, and histone hyperacetylation in HCT116 colon cancer cells [75].

##### Cardiovascular Protection

The consumption of broccoli sprouts or SFN could decrease oxidative stress in cardiovascular and kidney tissues. This might be due to the decreased protein nitrosation and high levels of glutathione, glutathione peroxidase, and glutathione reductase [57]. In addition, the endothelial relaxation of the aorta was enhanced, the quantity of activated macrophages was decreased, and the blood pressure remained normal. Thus, the feeding of isothiocyanates could improve the ventricular function and also decrease the myocardial size and cardiomyocyte apoptosis, thus preventing the chronic inflammation of cardiovascular tissues [76]. Among the numerous compounds contained in cruciferous vegetables, there are also those with cardioprotective potential. One of them is l-sulforaphane (LSF), which is formed from a glucoraphanin precursor by hydrolysis. In addition, due to its antioxidant and anti-inflammatory properties, it has a beneficial effect on the cardiovascular system, which has been confirmed by both in vitro and animal studies. Mirmiran et al. [77] have shown that broccoli extract and other products with a high GR content reduce the plasma low-density lipoprotein (LDL) cholesterol, but the mechanism of this interaction is not yet known.

##### Protection of the Central Nervous System

Similar to cardiovascular and carcinogenesis disease, chronic inflammation and oxidative stress are the main cause of pathogenesis of the central nervous system. The administration of SFN could decrease the infarct size, brain edema, and cortical apoptosis, reducing the inflammation and tissue damage of the central nervous system. Mostly, the neuroprotective impacts of SFN are due to the activation of the transcription factor Nrf2 and the upregulation of different target genes [58]. Interestingly, SFN administration also has a positive impact on developmental disorders associated with withdrawal and difficulties in establishing social relations. It is believed that this may be related to a reduction in the action of reactive oxygen species, whose significantly elevated levels are observed in the course of this type of disease, and their consequences include the damage of many important brain areas’ neurons. This is also confirmed by in vitro research on mouse cortical neurons, in which the addition of SFN reduced the damage induced by high concentrations of substances that mimic the pathomechanism of autism spectrum disorders. Other reports indicate that a diet rich in vegetables containing isothiocyanates is beneficial to schizophrenic patients, but more extensive research is needed to fully understand the mechanism that regulates these effects [78].

##### Protection against Neuropathy 

SFN was also found to be effective in improving renal performance and reducing pathological changes in glomerulus. The velocity of neuron conduction, blood flow, and pain behavior were significantly improved to alleviate different metabolic disorders and improve protection against renal damage [53].

##### Significance of Glucosinolates in Response to Fungi, Bacteria, and Microorganisms 

It is evident that the GSLs in *Brassicaceae* may play a significant role in the defence of the plant against fungal infection. However, the changes in disease resistance may depend on both the fungal pathogen species and the composition of the GSLs in the plant. This suggests that resistance against both fungi was due to a combination of the direct toxicity effect of GSLs and the activation of systemic acquired resistance. There is a strong interest in altering the levels of specific GSLs in order to obtain certain GSLs with desirable properties in terms of flavor; cancer prevention; and, in particular, pathogen protection [60]. However, the changes in disease resistance are also dependent on the pattern and effectiveness of the pathogens. Many reports confirm that the isothiocyanates and extracts from cruciferous plants inhibit the growth of pathogenic fungi. The antifungal potential is demonstrated by mustard oils, as well as volatile compounds contained in the roots and other brassicaceae tissues. It should be emphasized, however, that some studies indicate that glucosinolates do not exhibit fungicidal activity, and only the products of their hydrolysis have such properties. The level of toxicity to fungi varies with the structure of the compound. It is much higher in the case of volatile substances. As for the antifungial mechanism of GSLs, three pathways are considered: blocking phosphorylation, inhibiting electron transport, and ATP synthesis [64]. In turn, the products of hydrolysis of alkyl or aryl glucosinolates block the activity of bacteria. This was confirmed for the Salmonella typhinurium example. For instance, benzyl isothiocyanate has bactericidal and fungicidal properties and has proven effective in combating respiratory and urinary tract infections. However, the described properties are not the same for all microorganisms; Gram-negative bacteria are less sensitive to the action of GSL derivatives. Some organisms use a defence mechanism that blocks the action of key compounds. The well-known SFN allows for the effective control of Helicobacter pylori, the presence of which significantly increases the risk of gastrointestinal cancer. According to many authors, the mechanism of action of the described properties of GSLs and their derivatives is based on the inactivation of intracellular enzymes crucial for the pathogen functioning [79]. GSL hydrolysis products are also effective natural insecticides. Their advantage is low toxicity, quick decomposition, and efficiency in eliminating nematodes or flying insects. Thanks to the rapid release of these compounds into the soil, they can effectively inhibit the development of various species of insects. The mechanism that probably underlies the described effects is the inhibition of the activity of thiol groups of key enzymes, or the blocking of electron transport and ATP synthesis [65].

##### Benefits for Diabetic Patients 

A random double-blind placebo study in 103 Scandinavian patients diagnosed with type 2 diabetes mellitus showed that the daily consumption of broccoli extract for 12 weeks significantly reduces glucose and HbA1C levels. The most spectacular effect was observed in obese patients with high insulin resistance. Closer research by Iranian scientists has shown that the daily consumption of broccoli extract powder for 4 weeks reduces the plasma insulin levels in patients with the same type of diabetes. In addition, the beneficial effects of broccoli extracts have been demonstrated to minimize diabetes-related complications as well [80].

##### Benefits in the Skin Problems 

There are also reports of the potential beneficial effects of cruciferous vegetable consumption on skin problems. In five volunteers with epidermolysis bullosa simplex who were given daily broccoli extract, the alleviation of skin lesions and induced expressions of keratins 16 and 6 were observed. It has also been suggested that products rich in SFN can reduce the risk of skin lesions caused by UV radiation, especially in high-risk patients. Nevertheless, further research is necessary to confirm the therapeutic potential of cruciferous plants in skin diseases [81].

### 2.2. Organosulfur Compounds (OSCs)

#### 2.2.1. Chemical Structure and Biodegradation 

Alliaceous vegetables are considered to be the rich source of a large variety of beneficial bioactive compounds, such as oligosaccharides, arginine, flavonols, and OSCs. Alliaceous vegetables belong to the *Allium* plant family. *Allium* is a Latin word used for garlic, while the meaning of *Allium sativum* is cultivated garlic. The genus *Allium* belongs to the monocot flowering plants, which include almost 900 different species, such as cultivated onions, garlic, shallots, leeks, scallions, and chives [82]. The leaves and bulbs of onion have been used for culinary purposes because of their large variety of flavors and textures. Shallots, with less pungent flavors, are closely related to onions and are normally utilized in different cooked or pickled dishes. Chives are characterized by their green stalks with mild onion flavors, while leeks are distinguished by their leafy sheaths and slight onion flavor [83]. In general, the leaves and bulbs of these alliaceous vegetables have gained much importance due to their culinary purposes as well as for their health benefits. Ancient literature from China, Rome, India, Greece, and Egypt cited the medicinal and therapeutical applications of these vegetables [84]. Much of the studies focused on the health benefits of their sulfur-containing components because these OSCs have many interesting health benefits, including their anti-cancer properties. The sulfur-containing compounds in alliaceous vegetables are largely derived from the precursors γ-glutamyl-S-alk(en)yl-L-cysteines and S-alk(en)yl-L-cysteine sulfoxides. The active substance allicin (diallylthiosulfate) is responsible for the typical pungent smell and therapeutic properties. The OSC compounds are mainly responsible for the characteristic flavor and odor and comprise almost 0.5% and 1% of the dry weight of onions and garlic, respectively [85,86]. Many studies have highlighted the anti-cancer mechanisms of several preparations, extracts, and sulfur products of alliaceous vegetables, including redox modification, antimicrobial activity, and the decreased bioactivation of carcinogens. Therefore, Allium vegetables and their substances have gained importance in effectively regulating the biological processes of carcinogenesis to modify or reduce cancer risk [73,83]. 

**Figure 3:**
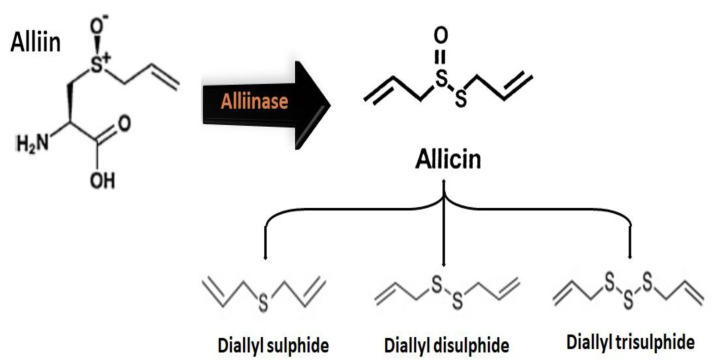
Organosulfur compounds and possible reactions of allicin.

Allium plants are consumed worldwide as vegetables, seasonings, and spices. Several types of Allium products, such as pastes, oils, salts, powders, and flakes, have been used in industries and homes as food seasonings and flavorings. These products can be prepared by different methods, including boiling, frying, baking, and drying. The differences in preparation methods can produce different amounts of bioactive sulfur compounds [87,88]. These bioactive sulfur compounds are mostly obtained when chewing, crushing, or cutting have damaged the alliaceous vegetables, facilitating a reaction between the enzymes and substrates (Figure 3). During the disruption of the cellular tissues of these vegetables, alliin is converted to allicin, which rapidly transforms into diallyl sulphide, diallyl disulfide, or diallyl trisulphide through the action of the enzyme alliinase. These intermediate products in alliaceous vegetables are considered health-promoting bioactive components [89,90]. 

Alliaceous vegetables are processed to different products such as dietary supplements, oil macerates, essential oils, powders, and extracts. These are available in numerous fresh and dehydrated products and are utilized in a wide range of medicine and dishes [91]. The dehydrated products are produced by the dehydration of alliaceous plants in hot air tunnels. *Allium* oil is produced by the distillation of minced vegetables (especially garlic). Juice extraction from milled alliaceous vegetables is conducted either by decantation or pressing. Allium extracts are extracted by the alcoholic extraction (methanol and ethanol) of raw materials. During extraction, nitrogen gas or liquid nitrogen can be added to isolate, concentrate, and trap the volatile compounds in the products [92]. The temperature, processing type, pH, time, and food matrix can affect the alliinase activity and thus regulate the stability of the bioactive compounds in allium products. Heating results in the inactivation of the alliinase enzyme, thus decreasing the amount of allicin metabolites in products. A large amount of allicin is lost during the processing of alliaceous vegetables [93]. This reduction in OSCs is linked to a decrease in flavor and reduction in the antimicrobial and anticancer potential of Allium products [94]. The OSCs in Allium plants are thermally unstable and tend to be lost during pasteurization, sterilization, cooking, and drying [82]. High-temperature processing, such as boiling and autoclaving, exerts negative effects on the bioavailability of organosulfur compounds [95,96]. High-pressure processing (HPP) can also decrease the allinase activity, thereby reducing the antimicrobial, anticancer, and antioxidative properties of Allium plants [97]. On the contrary, some studies suggested that HPP with a lower pressure (150 to 300 MPa) can be effective in modulating pyruvate formation due to the enzymatic activities linked with the formation of various sulfur compounds [98,99]. Moreover, freeze-drying and infrared-drying technologies can also reduce the thermal degradation of organosulfur compounds in Allium plants [100]. Similarly, non-conventional extraction techniques, such as microwave-assisted extraction (MAE) and pressurized liquid extraction (PLE), appear less suitable for OSC extraction due to their negative thermal effects, while supercritical fluid extraction (SFE) is considered to be an effective alternative non-conventional technique for the efficient extraction of essential oils and sulfur compounds from *Allium* sp [82]. Therefore, suitable and effective processing methods must be employed to retain bioactive sulfur compounds in products.

Bioactive sulfur compounds may undergo undesirable degradation during processing—in particular, conventional thermal techniques may negatively impact the bioavailability and physiological characteristics of *Allium* products. As stated earlier, heat treatment inactivates alliinase, thereby reducing the bioactive contents of the *Allium* product and diminishing the anticancer effects of these vegetable [101]. Additionally, heat may also alter the appearance of vegetables and result in unacceptable quality losses [102]. There is a general consensus that controlling the enzymatic activity in order to achieve the desired health benefits is of vital importance [103,104]. Thus, numerous research efforts have been made to develop innovative technologies to efficiently regulate the enzyme inactivation with the maximum retention of bioactive compounds. New processing technologies, such as using high pressure, high-voltage electrical discharges, pulsed electric fields, enzymology, and ultrasounds, have the potential to effectively retain the bioactive sulfur compounds in treated food products. However, the current knowledge about the retention of bioactive sulfur compounds in alliaceous vegetables after traditional processing is scarce, and there is also lack of information about the effects of the non-conventional processing of these vegetables [105,106]. Thus, there is still a need to conduct detailed studies evaluating the effect of non-conventional processing on health-promoting foods containing sulfur compounds.

#### 2.2.2. Health Benefits of Organosulfur Compounds 

Sulfur comprises approximately 1% of the dry weight of garlic, and the sulfur-rich compounds found in garlic correspond to its health benefits, especially in terms of cancer prevention and therapy [83]. The *S*-alk(en)yl-l-cysteine sulfoxides (ACSOs) content in fresh garlic is 3–14 mg/g of the fresh weight [107]. Among ACSOs, alliin (*S*-allylcysteine sulfoxide) is the major SACSO found in garlic. Another garlic compound that has been proven to induce apoptosis in tumor cells is the alliin thiosulfinate metabolite, allicin [108,109]. The anticancer mechanism of action for the active compounds could be indirect (via enhancing the immune system) or direct, via the conversion of allyl sulfides into sulfane sulfur in tumor cells, influencing proliferative signals [110,111]. Their role in cancer has been widely studied and the mechanisms of action have been proposed. Active sulfur-containing compounds induce apoptosis; promote chemoprevention by the induction of xenobiotic-metabolizing enzymes; ameliorate the detoxification of carcinogens; and are involved in cell cycle arrest, as well as in the blocking of the metabolism of hydrocarbons and nitrosamines, scavenging free radicals, and modulating the enzymes responsible for DNA repair [112,113]. Most of the studies that aimed to characterize the role of garlic-derived compounds in cancer were conducted in cell cultures and animal models of cancers [114], but there were also several clinical studies. For example, for prostate cancer in a population-based study it was revealed that alliaceous vegetables influenced the incidence of prostate cancer, with males consuming the allium having a reduced risk of prostate cancer development [115,116]. Allin and allicin were also studied with regard to breast cancer cells. Modem et al. revealed that fresh garlic extract could affect the growth and morphology of breast adenocarcinoma MCF7 cells (a useful model for the in vitro study of breast cancer). The authors demonstrated that garlic alters the morphology of the tumor cell via the inhibition of the expression of various cell adhesion, cytoskeletal, and morphology-associated signaling molecules. Due to the changes in the MCF7 cells’ morphology, the loss of cell-to-cell contact [18] was observed. Additionally, the study of allin in combination with paclitaxel demonstrated the increased cytotoxic effect of the anti-cancer synthetic drug for breast cancer cells. Further, the research conducted in 2020 by Rosas-Gonzalez et al. proved, for the first time, that allicin exerts a strong effect as an inducer of cell death in senescent cells. The authors discussed the potential of allicin to decrease the cell viability and apoptosis (probably via the mitochondrial pathway) in triple-negative breast cancer HCC-70 cells. The human studies were conducted in France and Mexico. They revealed a 70% decrease in the risk of breast cancer both for pre- and postmenopausal women [117,118]. The results may be indeed overestimated by the authors, since only 12 participants notified that they have never consumed garlic. Allicin was also studied in the human hepatocellular carcinoma (HCC) cell lines. Zhou et al. demonstrated in cell lines and a xenograft tumor model that allicin acts synergistically with 5-fluorouracil, even in subcytotoxic doses in vitro [119]. Recently, allicin was also proved to suppress the malignant phenotype of cervical cancer based on a study involving the human cervical squamous cell carcinoma cell line (Table 2). The main mechanism underlying this phenomena was revealed to be connected to the abnormal activation of the anti-oxidant enzyme, nuclear factor erythroid 2-related factor 2 [120].

Allicin is highly unstable and is converted to lipid-soluble sulfides such as diallyl sulfide (DAS), diallyl disulfide (DADS), and diallyl trisulfide (DATS) [83]. Those sulfur-based components have also been investigated for their utility in cancer. DATS was revealed to have a role in increasing the histone acetylation, leading to the suppression of the oncogenic protein expression in human cancer cells for, i.e., glioblastoma [113]. In 2015, Wallace et al. demonstrated in an in vitro study on glioblastoma cells that, indeed, DATS reduced the tumor volume and mitotic index by preventing the cell cycle progression and oncogene expression [126]. Furthermore, a dose-dependent meta-analysis (21 studies that included 543,220 subjects) conducted in 2011 by Zhou et al. [43] revealed that the consumption of one garlic bulb per day reduced the incidence of gastric cancer. DATS was also studied in pharmacological doses of 2.5 and 5 μM/person for its role in breast cancer inhibition and chemoprevention. There was verified evidence that these dosages of DATS were not harmful for the normal human mammary epithelial cell line, proving DATS’ importance as an anti-cancer compound [127]. The mechanisms of DATS in that hormone-related cancer were revealed to be connected to the inhibition of estrogen receptor α expression [81], breast cancer stem cell and metastasis inhibition [128,129], and leptin-induced oncogenesis in the MCF-7 cell line, as well as in the basal-like SUM159 xenograft mice model [130]. Furthermore, the studies revealed that the Notch-1 signaling pathway was disrupted by DATS. DATS inhibits the expression of proteases from the group of disintegrin and metalloproteases (ADAMs), especially ADAM-10 and ADAM-17, in estrogen-dependent and estrogen-independent breast cancer cells [131]. For another cancer where the Notch-signaling pathway is upregulated—osteosarcoma—DATS also proved to be efficient. Li et al. treated the human osteosarcoma cell lines U2OS and SaOS-2 with DATS and observed the inhibition of cancer proliferation, invasion, and angiogenesis driven by the reduction in the Notch-1 expression. The decreased Notch-1 expression is further connected to the downregulation of vascular endothelial growth factor (VEGF) and matrix metalloproteinases’ (MMPs) gene expression. Additionally, DATS was examined for that cancer as a promising factor able to reverse drug resistance [132]. The evaluation of DATS’ in vivo potential in breast cancer deserves to be further investigated. In the research of Kalra et al. [133] and Shrotriyaet al. [134], DATS and DAS were also proven to have the ability to induce the apoptosis of the carcinogen-induced two-stage mouse model of skin tumor via targeting multiple cancer signaling pathways. Additionally, DATS was able to induce cell cycle arrest and apoptosis in human melanoma cells [135]. Still, the investigation of the beneficial role of these compounds in UV-induced skin cancers should be continued.

Also, garlic-derived water-soluble compounds, such as S-allyl cysteine (SAC) and S-allylmercaptocysteine (SAMC), were investigated with respect to cancer. By processing garlic, an aged garlic extracts rich in SAC (0.62 mg SAC/g product) and SAMC, among other things, could be obtained. SAC could be easily absorbed from the gastrointestinal tract in an unchanged form and have a 30 times lower toxicity than some other garlic bioactive components in studies conducted in animal models. SAC displays stronger antioxidant properties than fresh garlic, and it has strong anti-cancer properties not only at the early but also at the late stages of the disease. A thiol nucleophile group in SAC thiol is responsible for the scavenging of free radicals, as well as activating antioxidant enzymes such as catalase and glutathione peroxidase [80,136,137]. The studies involved distinct cancer cell lines—i.e., ovarian, prostate cancers, neuroblastoma, human gastric, colon adenocarcinoma, and hepatocellular carcinoma [16,138]. The observed effects were exerted by the suppression of metastasis, the modulation of the carcinogen metabolism, influencing the mitochondrial membrane polarization, decreasing carcinogen binding to DNA, and scavenging reactive oxygen and nitrogen species [16,78].

Some other epidemiological studies were poorly organized and did not reach statistical significance [139,140]. Moreover, most of the clinical studies were performed before 2010, as in the case of garlic’s potential for gastric cancer [43].

## 3. Conclusions

There is some evidence that natural sulfur-containing phytochemicals could display anticancer properties. The purported compounds and mechanisms of action have been identified. The observed effects seem to depend on the concentration and bioavailability of the compounds. The biggest challenge is to determine the criteria for the storage and preparation or processing of alliaceous and cruciferous vegetables. Furthermore, conducting a number of clinical trials on a significantly large number of volunteers to optimize doses and learning the factors determining the bioavailability is necessary. Finally, the safety and potential effectiveness of treatment in specific diseases should be assessed. A metanalysis of the available trustworthy clinical records should be performed.

## Figures and Tables

**Table 1:** Health-promoting role of Glucosinolate.

Glucosinolate	Type of Compound	Health Promoting Roles and Plant Protection	Reference
Sulforaphane	Isothiocyanates	Inhibition of transcription regulator (NF-κB), which is relevant against inflammation and for minimizing diabetes-related complications such as diabetic neuropathy.	[13,53]
		Mediation of cell cycle arrest and apoptosis; inhibition of the activity of histone deacetylase; and increasing histone acetylation, which leads to the enhancement of protection against carcinogenesis. Induction of cytotoxicity.	[54,55,56]
		Normalization of kidney genome and blood pressure owing to the decrease in oxidative stress in cardiovascular and kidney tissues.	[57]
		Decreasing infarct size, brain edema, and cortical apoptosis, reducing the inflammation and tissue damage of the central nervous system due to the activation of the transcription factor Nrf2, and the upregulation of different target genes.	[58]
		Reduction in the damage induced by high concentrations of substances that mimic the pathomechanism of autism spectrum disorders in mice models.	[59]
		Treatment of *Helicobacter pylori.*	[60]
		Reduces the risk of skin lesions caused by UV radiation, especially in high-risk patients.	[44]
Glucoiberin, Sinigrin, and Progoitrin		Suppressing agents, protection of human and animal cells against carcinogenesis owing to the induction of Phase II detoxification enzymes or the inhibition of Phase I enzymes.	[60,61,62,63]
Indole-3-Carbinol		Chemopreventive agent.	[13]
Benzyl Isothiocyanate		Bactericidal and fungicidal properties and has proven effective in combating respiratory and urinary tract infections.	[64]
		Chemopreventive agent.	[13]
Allyl Isothiocyanate, Allyl Thiocyanate, and Allyl Isocyanate		Effective natural insecticides, efficiency in eliminating nematodes or flying insects. Possible mechanisms: the inhibition of the activity of the thiol groups of key enzymes, or the blocking of electron transport and ATP synthesis.	[65]

**Table 2:** Metabolic role of different organo-sulfur compounds in the human body.

Organosulfur Compound	Type of Compound	Health Promoting Role	Reference
Alliin (S-Allylcysteine Sulfoxide)	Natural bioactive constituent, with general formula C6H11NO3S.	It modulates the generation of proinflammatory cytokines by increasing the expression of cytokine genes like IL-6, MCP-1, and EGR-1. It also shows strong antioxidant and radical-scavenging properties. Alliin has also been found to boost the immune response in blood.	[4,31]
Allicin	Thiosulfinate and also the precursor of various sulfur-containing compounds, with the general formula C6H10OS2.	Allicin exhibits anti-cancer, anti-bacterial, anti-fungal, and anti-tumor activities. Allicin can inhibit the proliferation of tumor cells and can induce apoptosis in gastric cells by activating both the intrinsic and extrinsic pathways. Allicin shows anti-bacterial effects against a wide range of Gram-negative and Gram-positive bacteria (Staphylococcus, Escherichia, Klebsiella, Salmonella, Bacillus, Streptococcus, Proteus, and Clostridium). Moreover, allicin can also stimulate cytokine release, enhance immune resistance, and has anti-parasitic activity against several parasites.	[61,62,63]
Sulfenic Acid	First member of the family of organosulfur oxoacids, with the general formula RSOH.	Upon the chopping, damage, chewing, or crushing of Allium plants, the enzyme alliinase catalyzes the decomposition of alliin into short-lived and unstable sulfenic acid. It is thought to be responsible for antioxidant activities.	[32]
Diallyl Sulfide (DAS)	Derived bioactive compound which is a lipophilic thioether, with the general formula C6H10S.	Diallyl sulfide can boost the detoxification functions of liver cells, preventing symptoms of inflammation. It significantly enhances the production of the enzyme glutathione S-transferase (GST), which binds the electrophilic toxins inside the cell. DAS can inhibit the activation of nicotine-derived nitrosamine ketone (NNK), which is related to carcinogenesis. The preventive treatment with DAS also decreases the acetaminophen-induced hepatotoxicity and nephrotoxicity, indicating that it can decrease liver damage induced by drugs. It is also found to be effective against cardiovascular disease and colon cancer.	[35,36]
Diallyl Disulfide (DADS)	Derived organosulfur compound with the general formula C6H10S2.	Diallyl disulfide has multitargeted anti-carcinogenic activities, by (1) promoting carcinogen metabolism, (2) retarding the progression of the cell cycle, (3) inhibiting the proliferation of cells and inducing apoptosis. Moreover, diallyl disulfide also inhibit histone deacetylase activities, which have a therapeutic effect to stop cancer, since it can modulate histone hyper-acetylation and can reactivate tumor suppressor genes involved in cancer progression. Besides these benefits, it can induce allergens in *Allium* plants.	[35,36]
Allitridin or Diallyl *tri*-Sulfide (DATS)	Derived organosulfur compound with the formula C6H10S3.	Diallyl trisulfide have several health-promoting benefits, including having anti-cancer properties, being an antiviral immune booster, causing an increase in the reactive oxygen species (ROS) level, causing platelet aggregation, causing a decrease in blood pressure, causing cholesterol reduction, and being helpful in the treatment of cardiac arrhythmias. It has been found to selectively kill the cancerous cells in the breast and prostate, leaving the healthy cells unharmed.	[39,41,121,122,123,124,125]
